# Host Platelets and, in Part, Neutrophils Mediate Lung Accumulation of Transfused UVB-Irradiated Human Platelets in a Mouse Model of Acute Lung Injury

**DOI:** 10.1371/journal.pone.0044829

**Published:** 2012-09-19

**Authors:** Xuan Chi, Li Zhi, Monique P. Gelderman, Jaroslav G. Vostal

**Affiliations:** Laboratory of Cellular Hematology, Division of Hematology, OBRR, Center for Biologics Evaluation and Research, Food and Drug Administration, Rockville, Maryland, United States of America; French National Centre for Scientific Research, France

## Abstract

We previously reported that ultraviolet light B (UVB)-treated human platelets (hPLTs) can cause acute lung injury (ALI) in a two-event SCID mouse model in which the predisposing event was Lipopolysaccharide (LPS) injection and the second event was infusion of UVB-treated hPLTs. To delineate contributions of host mouse platelets (mPLTs) and neutrophils in the pathogenesis of ALI in this mouse model, we depleted mPLTs or neutrophils and measured hPLT accumulation in the lung. We also assessed lung injury by protein content in bronchoalveolar lavage fluid (BALF). LPS injection followed by infusion of UVB-treated hPLTs resulted in sequestration of both mPLTs and hPLTs in the lungs of SCID mice, although the numbers of neutrophils in the lung were not significantly different from the control group. Depletion of mouse neutrophils caused only a mild reduction in UVB-hPLTs accumulation in the lungs and a mild reduction in protein content in BALF. In comparison, depletion of mPLTs almost completely abolished hPLTs accumulation in the lung and significantly reduced protein content in BALF. UVB-treated hPLTs bound to host mPLTs, but did not bind to neutrophils in the lung. Aspirin treatment of hPLTs *in vitro* abolished hPLT accumulation in the lung and protected mice from lung injury. Our data indicate that host mPLTs accumulated in the lungs in response to an inflammatory challenge and subsequently mediated the attachment of transfused UVB-hPLTs. Neutrophils also recruited a small percentage of platelets to the lung. These findings may help develop therapeutic strategies for ALI which could potentially result from transfusion of UV illuminated platelets.

## Introduction

Although platelets are transfused for their life-saving hemostatic benefits, they can be associated with substantial adverse events, such as sepsis, alloimmunization and transfusion-related acute lung injury (TRALI) [1]. Among these, TRALI has emerged in recent years as the leading cause of transfusion related mortality reported to FDA [2]. The cellular and molecular mechanisms of lung injury in TRALI are still poorly understood. Recent animal studies have supported a two-event model [3], [4], [5], [6] in which TRALI requires an immune priming event, most often inflammation, that causes priming of polymorphonuclear cells (PMNs) and activation of pulmonary endothelial cells. This is followed by a transfusion event that introduces biologically active mediators such as lipids and cytokines from stored blood products [5], [6], [7] or anti-HLA antibodies, or anti-granulocyte antibodies [3], [4], [7]. These biologically active mediators are able to activate the primed PMNs, resulting in pulmonary endothelial cell damage and a capillary leak which are the hallmarks of acute lung injury (ALI) [8].

UV light has been used on platelet transfusion products to activate chemically-mediated pathogen reduction (UVA/amotosalen HCl (S-59), Cerus Corp. and UVB/riboflavin, Navigant Corp.). *In vitro* studies have demonstrated multiple log reduction of pathogens in platelets after UV light exposure and chemical treatment, thus supporting the concept that pathogen reduction treatment could effectively reduce platelet transfusion-associated infections [9], [10]. However, data from a blinded, randomized, prospective clinical trial of pathogen reduced platelets (UVA/amotosalen HCl (S-59) in the US (the SPRINT trial) [11] has raised safety concerns for photochemical treatment of platelets. In the study, there was a statistically significant difference in the number of Acute Respiratory Distress Syndrome (ARDS) cases reported in UVA/S-59 treated platelet arm vs the control platelet arm. A retrospective reanalysis of pulmonary data on a smaller number of patients according to specific clinical criteria for ALI and ARDS by a panel of pulmonary physicians identified a total of 12 cases of ARDS in the UVA/S-59 arm and 5 cases in the control arm [11] . However this difference did not reach statistical significance and the issue whether pathogen reduced platelets can mediate or contribute to respiratory distress in transfused patients remains unresolved. It has been shown that UV illumination can damage cells and pathogen reduction processes (UVA/S59 and UV/Riboflavin) damage platelets as is evident from their reduced in vivo recovery and survival in circulation post treatment [12], [13]. This prompted us to ask the question whether UV damaged platelets could have contributed to the higher rate of ARDS in the treatment group. We recently reported that UVB treated hPLTs were sequestered in the lungs of LPS primed SCID mice and induced ALI [14]. In this follow-up study, we wanted to understand the cellular mechanisms and the sequence of events that lead to ALI, using the same SCID mouse model.

## Materials and Methods

### Ab and reagents

mAbs and reagents used for immunostaining include anti-human CD41 (HIP8) (ABBIOTEC, San Diego, CA), anti-mouse CD41(BD Bioscience, San Diego, CA), anti-Gr1(clone RB6-8C5) and matched isotype control (BD Pharmingen, San Jose, CA), anti-mouse GPIbα (Emfret Analytics, Germany), anti-GFP (Invitrogen, Carlsbad, CA), fluor 488-conjugated, goat anti-mouse IgG_1_ (Invitrogen, Carlsbad, CA), biotinylated goat anti-mouse IgG_1_ (SouthernBiotech, Birmingham, Alabama), vectastain ABC elite kit and DAB kit (Vector Laboratories Inc. Burlingame, CA), Hoechst 33342 (Invitrogen, Carlsbad, CA). All mAbs used for flow cytometry were purchased from BD Bioscience (San Diego, CA) unless otherwise specified. These include anti-human CD41-FITC (or PE, clone HIP8), anti-human CD62P-PE (clone AK-4), anti-mouse CD41 PE, anti-mouse GPIbα-FITC (Emfret Analytics, Germany), as well as matched isotype controls.

### Animals

SCID mice (on BALB/c background), 6 to 8 weeks old, were obtained from NCI/DCT. CD41-eYFP mice (on C57BL/6 background) were the generous gifts from Dr. Thomas Graf (Center for Genomic Regulation, Barcelona, Spain) [15]. LYS-eGFP mice (on C57BL/6 background) were the generous gifts from Dr. David Sacks (NIAID, Bethesda, MD) [16], [17] upon approval from Dr. Thomas Graf. Female SCID mice were crossed with male LYS-eGFP and CD41-eYFP mice respectively to obtain double heterozygous mice, which were then intercrossed to get double homozygous (SCID/SCID; LYS-eGFP^ki/ki^ and SCID/SCID;CD41-YFP^ki/ki^), thereafter referred to as SCID/LYS-eGFP and SCID/CD41-YFP mice. Genotypes of CD41-YFP mice [15], LYS-eGFP mice [16], and SCID mice [18] were determined as published, and PCR protocols are available upon request.

All mice were kept in a pathogen-free facility and animal protocols were in compliance with guidelines provided by the Center for Biologics Evaluation and Research Animal Research Advisory Committee.

### Platelet products

Leukoreduced hPLTs were collected by apheresis [14] at the NIH Division of Transfusion Medicine, under full institutional review board approval. Platelets were allowed to rest overnight on a Helmer platelet agitator (Helmer, Noblesville, IN) to reduce the mechanical activation induced by the collection protocol. Experiments were performed twenty-four hours after collection.

### UVB irradiation of hPLTs

UVB irradiation of hPLTs was performed as previously described [14]. The total UVB dose per sample was calculated to be 2.4 Joules/cm^2^. UVB energy was from two fluorescent lamps (UVP, Upland, CA).

### Thrombin receptor-activating peptide (TRAP) activation of hPLTs

TRAP was purchased from Sigma (St. Louis, MO). Twenty mL samples of platelet products were taken aseptically from the platelet bags and split in two 10 mL aliquots. One set of 10 mL aliquots was treated with TRAP (4 uM) and incubated at 37°C for 2–10 min. The other set of 10 mL aliquots were used as controls and were not treated with TRAP but otherwise processed in the same manner.

### Labeling of hPLTs with Orange CMTMR

The hPLT labeling procedures with CMTMR was adopted from published protocol [19]. Briefly, we collected platelets from the platelet-rich plasma by centrifugation at 834 g for 5 min, washed them in platelet Wash Buffer (140 mM NaCl, 5 mM KCl, 12 mM trisodium citrate, 10 mM glucose, 12.5 mM sucrose, 1 µg/ml prostaglandin E1, pH 6.0) and resuspended in platelet Resuspension Buffer (10 mM HEPES, 140 mM NaCl, 3 mM KCl, 0.5 mM MgCl2, 10 mM glucose, and 0.5 mM NaHCO3, pH 7.4). CMTMR dye (Invitrogen, Carlsbad CA) was added to the resuspended platelets to a final concentration of 2.5 mM and incubated at 37°C for 30 min. Platelets were again washed by platelet Wash Buffer before resuspended in 500 uL of Resuspension Buffer, ready for a final cell count and injection.

### Cell counts

Complete blood counts were performed on a clinical hematology analyzer (Pentra 60+, ABX Diagnostics, Irvine, CA) or a Cell Dyn 3700 (Abbott Diagnostics, Santa Clara, California). Concentrations of apheresis platelet units were between 3.2×10^5^/uL to 1.7×10^6^/uL. For the final cell count before injection, concentrated hPLTs were diluted 1∶10 in plasma to bring the final platelet count within the range of the instrument.

### Transfusion of hPLTs and mouse whole blood collection

hPLTs (∼1×10^9^ platelets in 100-µL PBS or platelet Resuspension Buffer) were infused into mice via tail vein as previously described [14], [20]. Mouse whole blood was collected via tail vein bleeds.

### Lipopolysaccharides (LPS) administration

SCID mice were injected intravenously with 3 mg/kg of LPS (diluted in 100 µl PBS) from *Escherichia coli* 0111:B4 (Sigma, St. Louis, MO), or an equal volume of PBS two hours prior to injection of platelets.

### Bronchoalveolar lavage fluid (BALF) collection and assay for determining protein concentration in BALF

Mice were sacrificed one hour after platelet injection, and the tracheas were exposed and cannulated with an 18 gauge blunt needle. Lungs were immediately lavaged with 1 ml of PBS three times for a total infusion of 3 ml and a final recovery of 2.5 ml which were placed on ice. The lavage procedure was completed within 5 min post mortem. The lavage fluid was centrifuged at 200× g for 15 minutes at 4°C and the supernatants were analyzed for protein content by QuantiPro BCA Assay Kit (Sigma, St. Louis, MO) according to manufacturer's instructions.

### Antibody staining of lung sections

For Gr-1 mAb immunohistochemistry experiments, the lungs of euthanized mice were inflated by intratracheal injection of cold 4% PFA (1 mL). One lobe of the lung was tied off at the bronchus, excised and the entire lobe was submerged in 4% PFA and fixed overnight at 4°C. Lungs were washed once for 5 min in PBS, placed into 70% ethanol and sent for paraffin embedding and sectioning (Histoserv, Rockville, MD). Lung sections (5 µm) were used for anti-Gr-1 mAb staining, following published protocol [4] and imaged with a Nikon Eclipse E800 microscope (Nikon Co., Ltd., Tokyo, Japan). For staining with anti-hCD-41 antibodies, mouse lungs were inflated by intratracheal injection with a 1∶1 mixture of OCT compound/PBS (1 mL). One lobe of the lung was tied off at the bronchus, removed and snap-frozen in OCT in an ethanol dry-ice bath, and sectioned at 10 µm. Cryosections were post-fixed with Methanol (10 min, 4°C), blocked in 3% goat serum (Sigma) with 1% BSA in PBT at room temperature for 1 hr. The tissue sections were incubated with monoclonal mouse anti-human CD41 (1∶100) or GFP (1∶1000) antibodies overnight in 3% goat serum/PBT. An Alexa Fluor 568-conjugated, goat anti-mouse IgG_1_ (1∶600) was used as the secondary antibody for the anti-hCD41 antibodies. An Alexa Fluor 488-conjugated, goat anti-rabbit IgG was used as the secondary antibody for anti-GFP antibody. All sections were stained with Hoechst 33342 (Vector Laboratories, Burlingame, CA), mounted in VECTASHIELD (Vector Laboratories, Burlingame, CA), and photographed using a Zeiss LSM710 confocal microscope, with a 63×/NA1.4 Plan-Apochromat oil objective and 100×/NA1.4 Plan-Apochromat oil objective (Carl Zeiss Inc, Germany). Three serial lung sections from at least five mice of each treatment group were used for analysis. We confirmed, through immunofluorescence studies, that the anti-hCD41 antibodies we used to detect human platelets in the lung are specific to hPLTs (Figure S1-A), and do not cross-react with mPLTs (Figure S1-C).

### Neutrophil and platelet depletion

Neutrophil depletion was accomplished following an established protocol [4] by using a rat anti-mouse mAb against the neutrophil maturation antigen, Gr-1. Gr-1 mAb (250 µg, i.p.) was administered 24 hours before planned experiments. Platelet depletion was accomplished by using a mixture of purified rat monoclonal antibodies directed against mouse platelet GPIbα (CD42b), following manufacturer's recommendations (Emfret Analytics, Würzburg, Germany). GPIbα Ab (2 mg/kg, i.v.) was administered 4 hours before planned experiments. Neutrophil depletion (>95%) and platelet depletion (>95%) were both confirmed microscopically on multiple blood smears. Isotype control Abs were used in both depletion protocols.

### Aspirin experiments

Aspirin tablets (ASA, 325 mg) were crushed, dissolved in DMSO (100 mg/ml), and diluted in PBS for in vitro treatment. In vitro aspirin treatment (0.4 mg/ml) or vehicle control (DMSO) were delivered to platelet-rich plasma (PRP) 30 minutes prior to UVB illumination.

### Platelet aggregation studies

Platelet aggregation in PRP was measured on a Bio/Data PAP8E aggregometer (Horsham, PA), according to manufacturer's instructions, as a percentage change in light optical density from baseline (set to 0%) as compared with a reference platelet-poor plasma (PPP) sample. TRAP was used as agonists, at 20 µM. The Maximum Aggregation (MA) and Dis-Aggregation (DA) was monitored for the duration of 10 min.

### Flow cytometric analysis

Mouse whole blood or apheresis human platelets were incubated with the antibodies for 20 minutes at room temperature in the dark, and then cells were washed once (5 min at 1000× *g*, with 1 µg/ml prostaglandin E1) and resuspended in 500 mL of PBS containing 0.3% bovine serum albumin. Flow cytometry was performed on a flow cytometer equipped with its accompanying software (FACSCalibur and CellQuestPro, respectively, Becton Dickinson, San Jose, CA). Flow cytometric data was analyzed using FlowJo (version 7.6.3).

### Image analysis

Quantitative measure for the fluorescence signal (number of pixels in the selected channel) was obtained using Adobe Photoshop CS3, as previously described [14]. Three serial lung sections per mouse from at least five mice of each treatment group were used for analysis. Mean and standard deviation (SD) were calculated for each treatment group.

### Confocal imaging of the lung

The organs were dissected into PBS, immediately mounted onto glass slides with Secure-Seal spacers (Invitrogen, Carlsbad, CA) and imaged under a Zeiss LSM710 confocal microscope, with a 40×/NA1.0 Plan-Apochromat water objective (Carl Zeiss Inc, Jena, Germany). Alternatively, the organs were embedded into 4% low melting-point agarose and vibrotome (50 uM) sections were prepared. The sections were mounted onto glass slides with Secure-Seal spacer and imaged directly under Zeiss LSM710 confocal microscope. A single confocal image or a stack of confocal images were collected. For image stacks, a maximum intensity projection was produced on Zeiss Zen software (version 2.1).

### Statistical Analysis

A student's *t*-test was performed for statistical analysis with Windows Excel program with statistical significance set at *p*<0.05.

## Results

### TRAP (4 µM) activated hPLTs are not sequestered in the lung of LPS-primed SCID mice

**Figure 1:**
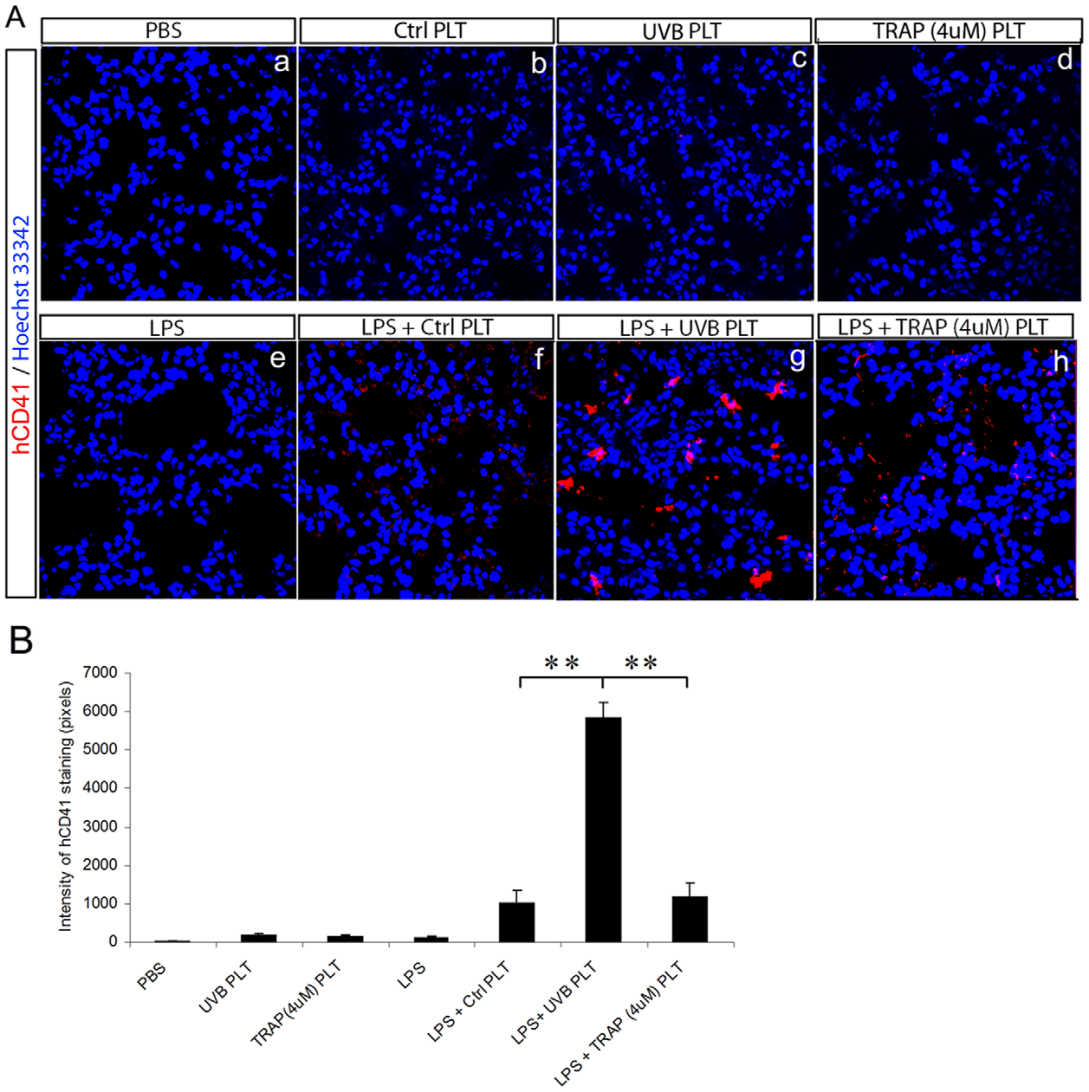
UVB treated hPLTs sequestered in the lungs of LPS primed SCID mice whereas TRAP (4 µM) activated hPLTs did not. **A:** Confocal images of lung sections of healthy (a to d) and septic (e to h) SCID mice infused with control human platelets (Ctrl PLT), UVB-treated human platelets (UVB PLT) or TRAP (4 uM) activated human platelets (Representative images from five independent experiments). In healthy mice, there were few hPLTs in the lung in any treatment group (a to d). Sequestration of significant number of hPLTs in the lung of LPS primed mice infused with UVB treated hPLTs was observed (g), whereas a much lower numbers of Ctrl PLT (f) or TRAP-activated hPLTs (h) were sequestered in the lung. Sections were stained with anti-hCD41 antibodies (red). Blue fluorescence represents Hoechst33342 stained nuclei. All images were taken using a Zeiss 710 laser scanning confocal microscope, with a 63×/NA1.4 Plan-Apochromat oil objective. **B**: Quantification of the fluorescence intensity of anti-hCD41 antibody staining on mouse lung sections. Data are expressed as means ± SD; n = 5. ** p<0.01.

We previously reported that UVB- treated hPLTs were sequestered in the lungs of LPS-primed mice and mediated ALI but this did not occur in healthy (non-LPS treated) mice [14]. This finding was again confirmed in the current study (Figure 1A-a to c and e to g) where we investigated the underlying mechanism of platelet accumulation in the lung and subsequent ALI. UVB treatment induces the expression of P-selectin, an activated platelet marker, in 35.9% of platelets which is higher than the expression of 17.8% on control platelets [14]. We tested whether P-selectin was the molecule that mediated the sequestration of UVB exposed platelets in the lung. hPLTs were treated with 4 µM of TRAP, which resulted in P-selectin expression on approximately 50% of platelets (data not shown). When these platelets were injected into LPS-primed SCID mice, the number of hPLTs in the lung (Figure 1A-h) was not significantly different from that of control PLT group (Figure 1A-f), and was much lower when compared to UVB-treated hPLT group (Figure 1A-g and 1B, P<0.01). We also treated hPLTs with 20 µM of TRAP, which resulted in >99% of platelets expressing P-selectin and visible platelet aggregation. When these platelets were injected into SCID mice, we observed a higher number of hPLTs in the lungs, but the number was still significantly lower than that of UVB-treated hPLTs group (data not shown). We did not pursue additional analysis of the tissue distribution of TRAP (20 µM) activated hPLTs in SCID mice, because cell count on these aggregated platelets is inaccurate and injecting these large aggregates via the tail vein is technically difficult.

### UVB-treated human platelet infusion does not result in neutrophil sequestration in the lungs of LPS-primed SCID mice

We studied whether neutrophils were sequestered in the lung concurrently with the accumulating platelets by using LYS-eGFP mice. The LYS-eGFP mice express enhanced green fluorescent protein (eGFP) under the control of the endogenous lysozyme M promoter, in which endogenous neutrophils are brightly fluorescent [16], [17]. We independently confirmed that GFP-expressing cells from these LYS-eGFP mice were of neutrophil origins that express Gr-1 (data not shown). The LYS-eGFP mice were crossed with SCID mice and the double homozygous SCID/LYS-eGFP mice were used in this study.

**Figure 2:**
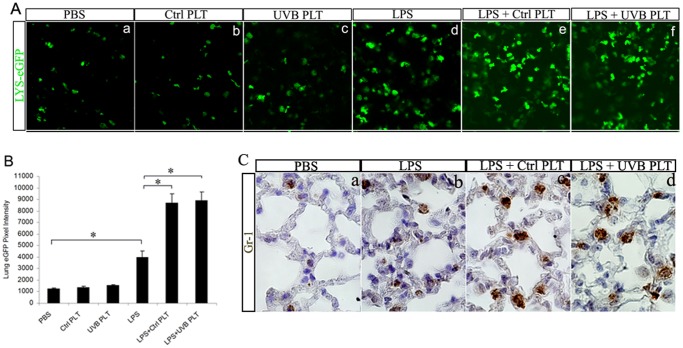
UVB treated hPLT infusion does not result in more neutrophil sequestration in the lungs of SCID mice as compared with control hPLTs. **A**: Confocal images of unfixed, vibratome sections of the lungs from SCID/LYS-eGFP mice. In these mice, neutrophils are labeled in green fluorescence. LPS primed mice were injected with LPS (i.v , 3 mg/kg). The composite images shown were representative of 5 independent experiments. All fluorescent images were taken with a Zeiss 710 laser scanning confocal microscope, with a Plan-Apo 40×/NA1.0 water objective. **B**: Quantification of LYS-eGFP fluorescence intensity on lung sections from 5 independent experiments. Data are expressed as means ± SD; n = 5. * p<0.05. **C**: Images of immunohistochemical staining on paraffin sections with anti-Gr-1 antibodies. The composite images shown were representative of 4 independent experiments. All transmitted light images were taken with a Nikon Eclipse E800 microscope (Nikon Co., Ltd., Tokyo, Japan), using a 20×/NA 0.45 Plan Fluor objective.

In healthy SCID/LYS-eGFP mice, numerous neutrophils were observed in the lung (Figure 2A-a). When Ctrl PLTs (Figure 2A-b) or UVB PLTs (Figure 2A-c) were injected intravenously into the mice, the number of neutrophils accumulated in the lung did not change significantly. There was a significant increase of neutrophils in the lungs when the mice were primed with LPS (Figure 2A-d and 2B), and even more neutrophils accumulated in the lungs when hPLTs (either control PLTs, Figure 2A-e or UVB-treated platelets, Figure 2A-f) were injected into mice primed with LPS. However, when the neutrophil count in the lungs of LPS+UVB-hPLT group (Figure 2A-f) was compared to LPS+Ctrl-PLT group (Figure 2A-e), the difference was not statistically significant (p>0.05, Figure 2B). This observation was confirmed by detection of neutrophils by immunohistochemistry with Gr-1 antibodies (Figure 2C). Histologically, lung sections from LPS+UVB PLT group also showed thickened aveolar septum and fibrin deposition (Figure 2C-d), as shown previously [14].

### Neutrophil depletion mildly reduces platelet retention in the lung of LPS primed mice

**Figure 3:**
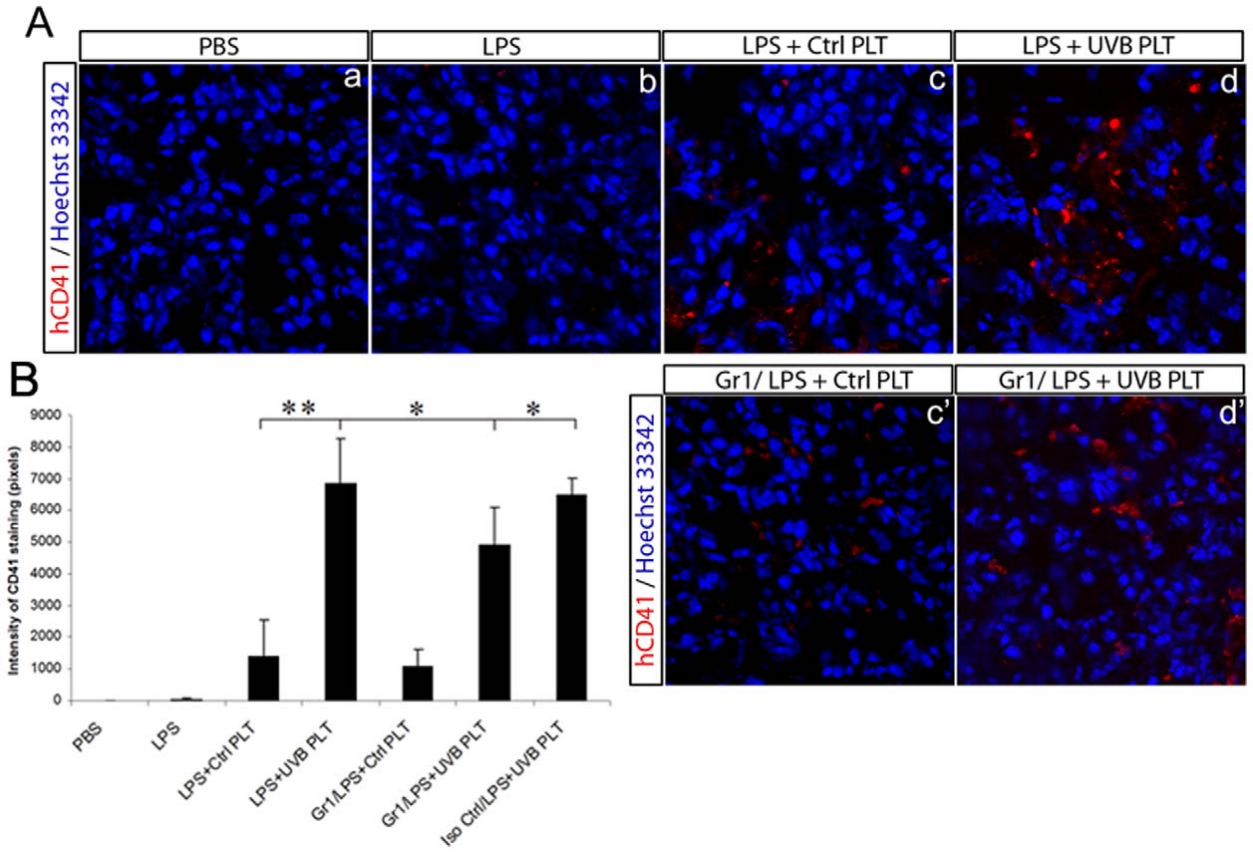
Neutrophil depletion reduced platelet sequestration in the lungs. SCID mouse were pretreated with either Gr-1 mAb (250 ug, i.p.) or isotype control (Iso Ctrl, i.p.) 24 hours before LPS administration (3 mg/kg, i.v.), followed by transfusion of control hPLTs or UVB-treated hPLTs. A: Confocal images of lung sections from SCID mice stained with anti-CD41antibodies (red). Blue fluorescence represents Hoechst33342 stained nuclei. The composite images shown were representative of 6 independent experiments. All images were taken using a Zeiss 710 laser scanning confocal microscope, with a 63×/NA1.4 Plan-Apochromat oil objective. B: Quantification of fluorescence intensity of anti-CD41 antibody staining on mouse lung sections. Data are expressed as means ± SD; n = 6 .* p<0.05; ** p<0.01.

We tested whether neutrophil depletion could abolish platelet accumulation in the lung. Neutrophils were depleted with Gr-1 mAb prior to LPS and platelet injections. When neutrophils were depleted, UVB-hPLT accumulation in the lung was not completely abolished, but was reduced by 20% on average as compared with the non-depleted group (Figure 3A-d′ vs d) or the isotype control treated group, and this difference was statistically significant (P<0.05, Figure 3B).

### LPS injection followed by infusion of UVB-hPLTs results in sequestration of both mouse and human platelets in the lung of SCID mice

**Figure 4:**
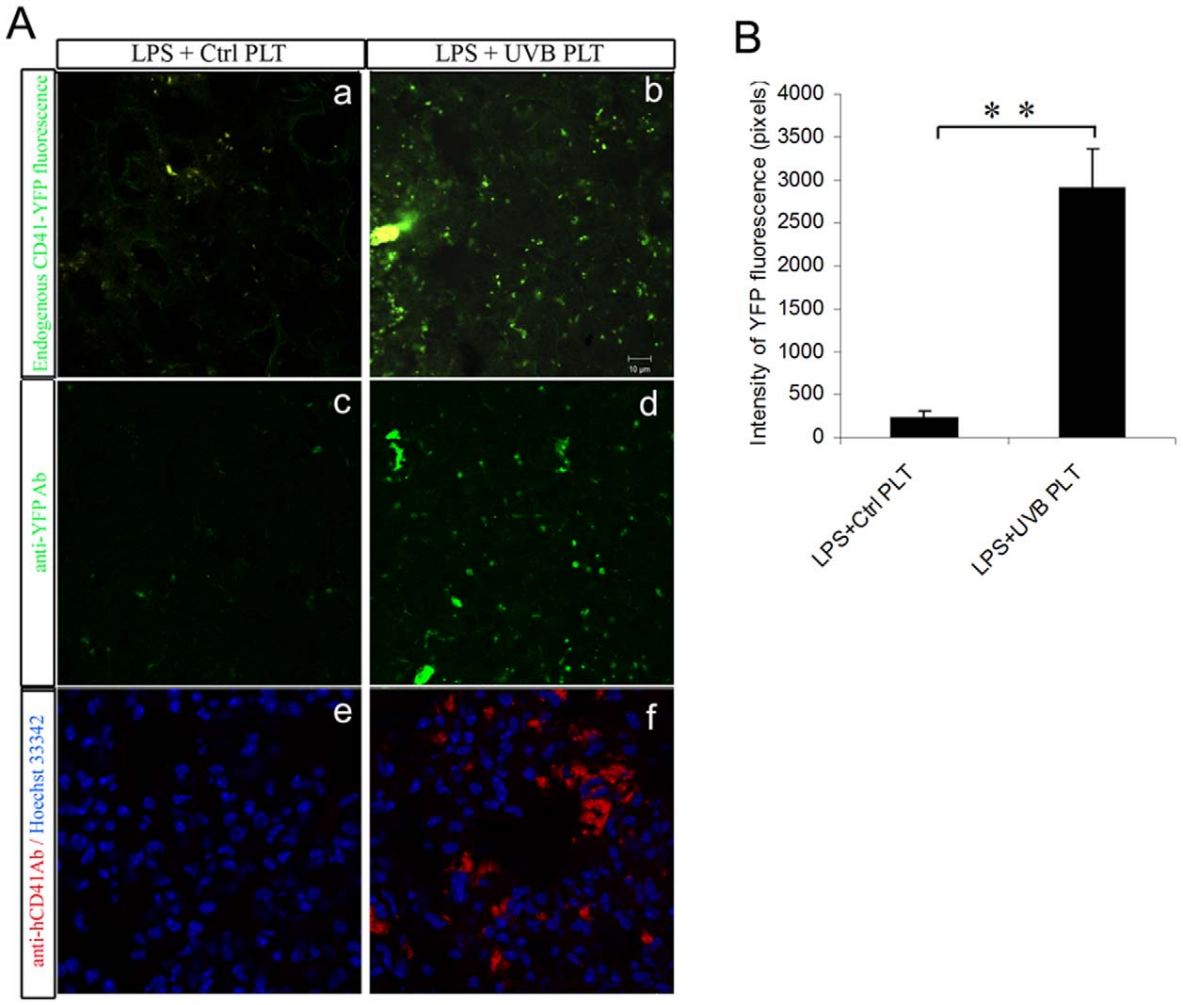
Infusion of UVB-treated hPLTs resulted in retention of both mouse and human PLTs in the lungs of LPS primed SCID mice. **A**: Confocal images of mouse lung sections. More mouse PLTs were sequestered in the lungs of SCID; CD41-YFP mice after UVB-treated human platelet injection (A-b,d), compared with the control (A-a, c), visualized by endogenous CD41-YFP fluorescence on fresh frozen sections (A-a, b) or anti-YFP antibody staining on fixed frozen sections (A-c, d). More hPLTs were sequestered in the lungs of the mice after UVB-treated hPLT injection (A-f), compared with the control (A-e), visualized by anti-hCD41 antibody staining on fixed frozen sections. The composite images shown were representative of 5 independent experiments. **B**: Quantification of pixel intensity of endogenous YFP fluorescence on SCID; CD41-YFP mouse lung sections. All images were taken using a Zeiss 710 laser scanning confocal microscope, with a 63×/NA1.4 Plan-Apochromat oil objective. Data are expressed as means ± SD; n = 5. **p<0.01.

As we have shown previously [14], significantly more hPLTs were accumulated in the lung of LPS primed mice in the UVB-PLT group as visualized by anti-hCD41 antibody staining (Figure 4-f vs e). In addition, significantly more mPLTs were also sequestered in the lungs of UVB-treated hPLT group (Figure 4A-a to d, Figure 4B). This was visualized by endogenous YFP expression from SCID/CD41-YFP mice (Figure 4A-b vs a), as well as by immunostaining of lung tissue with anti-YFP antibodies (Figure 4A-d vs c).

### Mouse platelet depletion abolishes both mouse and human platelet sequestration in the lungs of LPS primed mice

**Figure 5:**
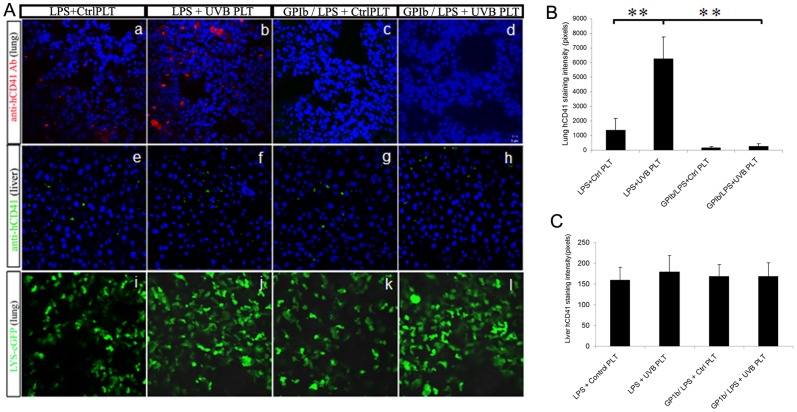
Mouse PLT depletion abolished hPLT sequestration in the lungs of LPS primed SCID mice. **A**: Confocal images of mouse lung or liver sections. GPIb antibody treatment abolished hPLT sequestration in the lungs of septic SCID mice (A-c,d vs. a, b), visualized by anti-hCD41 antibody staining. Yet GPIb antibody treatment did not abolish platelet accumulation in the liver (A-g, h vs e, f). Neutrophil count, visualized by endogenous LYS-eGFP fluorescence, in the lung was similar before and after PLT depletion (A-k vs i, l vs j). The composite images shown were representative of 5 independent experiments. All images were taken using a Zeiss 710 laser scanning confocal microscope, with a 63×/NA1.4 Plan-Apochromat oil objective. **B**: Quantification of fluorescence pixel intensity of anti-hCD41 antibody staining on SCID; CD41-YFP mouse lung sections shown in A-a to d. **C**: Quantification of fluorescence pixel intensity of anti-hCD41 antibody staining on SCID; CD41-YFP mouse liver sections shown in A-e to h. Data are expressed as means ± SD; n = 5. *p<0.05;**p<0.01.

SCID mice were pretreated with GPIb Ab (2 mg/kg, *i.v*.) 4 hours before LPS administration to deplete mPLTs. The GP1b antibodies do not react with human GPIb or other surface receptors on hPLTs (Figure S2). Both peripheral blood mPLTs and mPLTs in the lung were depleted 4 hours after receiving GPIb antibodies (Figure S3-c, d vs a, b and g, h vs e, f), as visualized by endogenous fluorescence of SCID/CD41-YFP mice. LPS primed animals, depleted of platelets, were then infused with UVB treated hPLTs or control hPLTs, and platelet accumulation in the lungs was evaluated 1 hour later. As a comparison hPLT accumulation in the liver were also evaluated. The depletion of mPLTs abolished hPLT sequestration in the lungs of LPS primed SCID mice (Fig. 5A-c, d vs. a, b) almost completely (−96%, Figure 5B), as visualized by anti-hCD41 antibody staining on fixed lung sections. However, the small number of hPLTs sequstered in the liver were still present after GPIb antibody treatment (Figure 5A-g, h vs e, f and Figure 5C), which indicates that GPIb antibodies do not deplete infused hPLTs nonspecifically and the presence of small number of hPLTs in the liver is not dependent on mPLTs. The neutrophil count in the lung, visualized by endogenous LYS-eGFP fluorescence, was similar before and after platelet depletion (Figure 5A-k, l vs i, j), indicating that mouse platelet depletion and the absence of hPLTs, does not prevent neutrophil sequestration in the lungs of LPS-primed mice.

### UVB-treated hPLTs bind to mPLTs in our two-event mouse model of ALI

**Figure 6:**
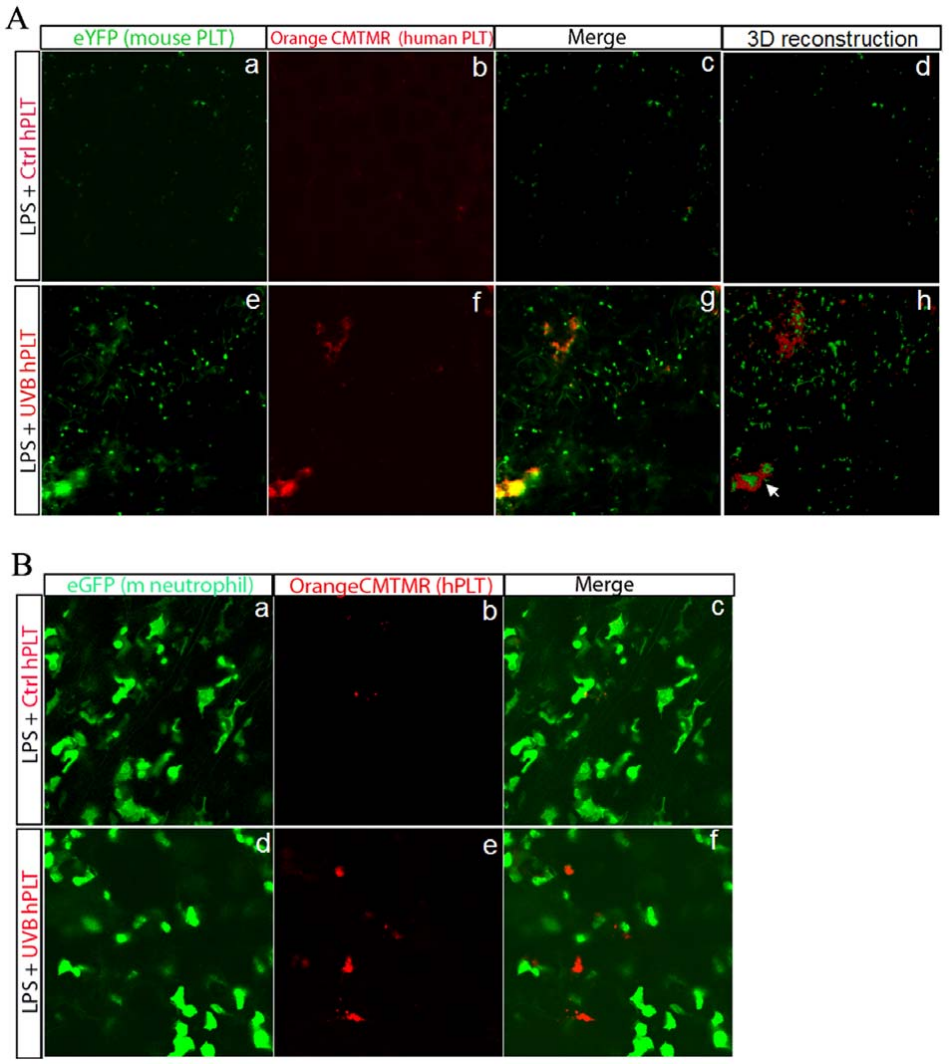
UVB-treated hPLT bind to host mouse PLTs, but not to neutrophils in the lung. **A**: Live images of whole-mount lungs from SCID/CD41-YFP mice, after LPS injection and injection of either Ctrl PLTs (a–d) or UVB PLTs (e–h). MPLTs were visualized by endogenous YFP expression (in green, a and e). Human PLTs were labeled with CMTMR (in red, b and f). Figure 6A-c, g show the merged images of mouse and human platelets in the lung. Figure 6A-d, h show 3D reconstructed images of a stack of 10 images (10 uM). When UVB-hPLTs were injected into SCID/CD41-YFP mice, hPLTs in the lung colocalize or in close proximity to mouse PLTs (Figure 6A-g, h). **B**: Confocal images of whole-mount lungs from SCID/LYS-eGFP mice, after LPS injection and injection of either Ctrl PLTs (a–c) or UVB PLTs (d–f). Mouse neutrophils were visualized by endogenous eGFP expression (in green, a and d). hPLTs were labeled with CMTMR (in red, b and e). c and f show the merged images of hPLTs and mouse neutrophils in the lung. When UVB-hPLTs were injected into SCID/LYS-eGFP mice, the majority of hPLTs in the lung don't bind to neutrophils (f).

To define the interaction between mPLTs and UVB-hPLTS, we labeled the UVB-hPLTS with fluorescent dye CMTMR and infused these into SCID/CD41-YFP mice. CMTMR is a vital dye and the labeling procedures do not cause additional platelet activation (Figure S4). In the lungs of LPS primed mice, the UVB-hPLT platelets colocalized with or were in close proximity to mPLTs (Figure 6A-f, g). Like we have shown previously [14] and in this paper, UVB-treated hPLTs often form aggregates in the lung (Figure 6A-f). On 3D reconstructed images, the human platelet aggregates clearly bound to mPLTs, and showed structures with intertwined layers of mouse and hPLTs (arrow in Figure 6A-h). This is in contrast with Ctrl-PLTs injected mouse lung, in which only a few single hPLTs were observed, and these hPLTs were randomly distributed (Figure 6A-b, c).

### UVB-treated hPLTs do not bind to pulmonary neutrophils in a two-event mouse model of ALI

We showed that when CMTMR labeled, UVB-hPLTs were injected into SCID/LYS-eGFP mice, the majority of hPLTs in the lung (mostly in the form of platelet aggregates) did not bind to neutrophils (Figure 6B-f).

### Aspirin treatment *in vitro* abolished platelet retention in the lung

**Figure 7:**
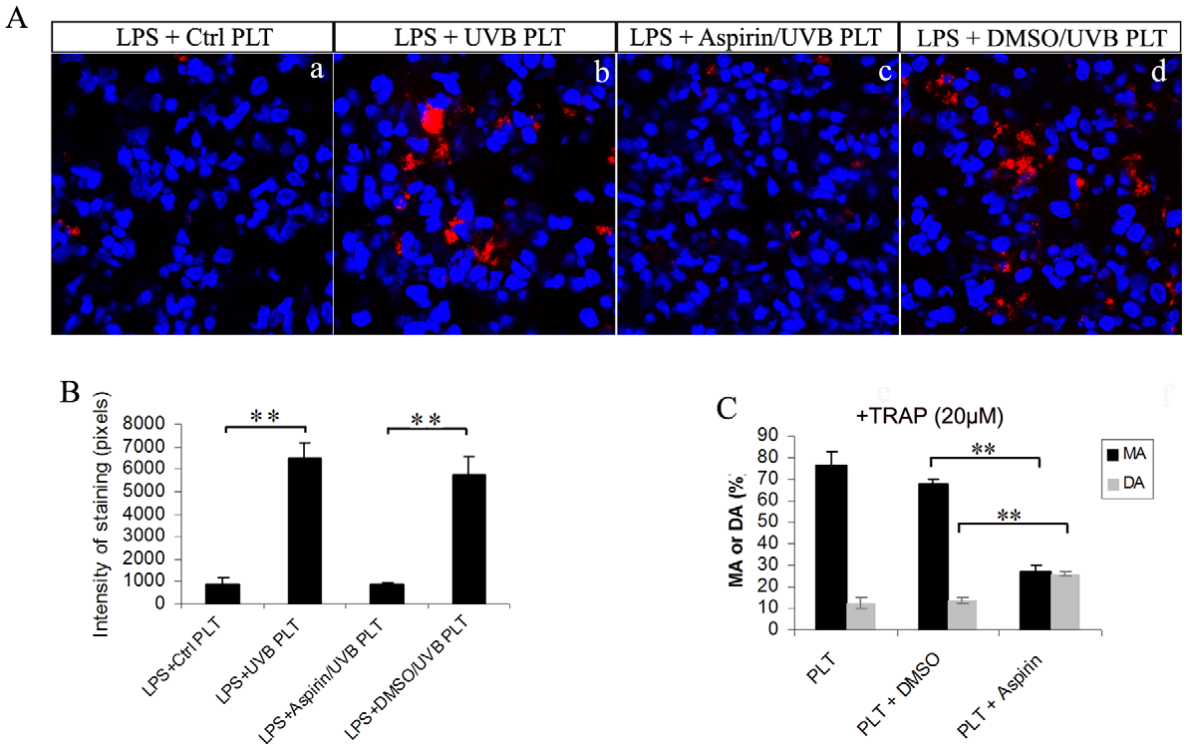
Treatment with aspirin *in vitro* prior to UV exposure abolished UVB-hPLT sequestration in the lung. **A**: Confocal images of SCID mouse lung sections. Pre-treatment with aspirin *in vitro* abolished UVB-induced hPLT sequestration in the lung (A-c), compared with vehicle control (A-d). All sections were stained with anti-hCD41 antibodies (red). Blue fluorescence represents TO-PRO-3 stained nuclei. The composite images shown were representative of 3 independent experiments. All images were taken using a Zeiss 710 laser scanning confocal microscope, with a 63×/NA1.4 Plan-Apochromat oil objective. **B**: Quantification of fluorescence intensity of anti-hCD41 antibody staining on mouse lung sections shown in A, n = 3. **C**: Aspirin treatment *in vitro* inhibited hPLTs aggregation induced by TRAP (20 µM). Maximum Aggregation (MA) was shown in solid black bar and Dis-Aggregation (DA) was shown in solid grey bar, n = 4. All data are expressed as means ± SD, ** p<0.01, * p<0.05.

We treated hPLTs with a cyclooxygenase inhibitor, acetylsalicylic acid (ASA, aspirin) or vehicle (DMSO) prior to UVB illumination. We chose a dose (0.4 mg/mL) that can inhibit TRAP induced hPLT aggregation *in vitro* (Figure 7C). Aspirin treatment *in vitro* almost completely eliminated hPLT accumulation in the lung (>95% on average, Figure 7A-c, 7B), compared with controls treated with vehicle (DMSO, Figure 7A-d), similar to the level of LPS+Ctrl PLT group (Figure 7A-b).

### Depletion of mouse neutrophils or platelets, or aspirin treatment of hPLTs *in vitro* all protected mice from pulmonary vascular permeability change

**Figure 8:**
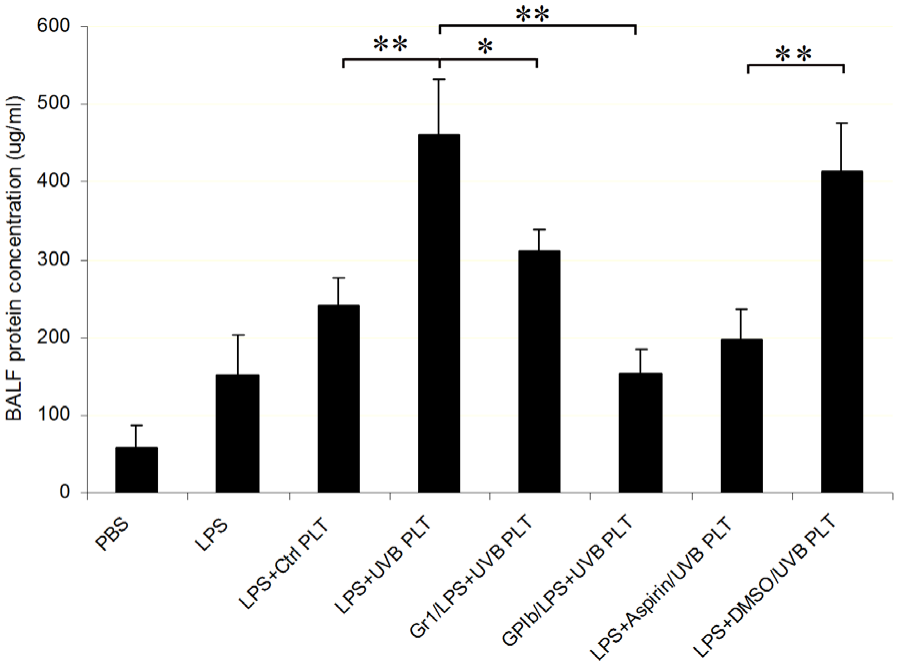
Depletion of mouse neutrophils or platelets and aspirin treatment prior to UV exposure all protected mice from pulmonary vascular permeability change. BALF protein concentration (ug/ml) was measured in different experimental conditions as indicated. N = 3 for LPS+Aspirin/UVB PLT and LPS+DMSO/UVB PLT, n = 5 for all other treatment groups. All data are expressed as means ± SD, ** p<0.01, * p<0.05.

To study whether depletion of neutrophils or platelets can protect SCID mice from ALI, BALF was collected from mice with or without pretreatment with Gr-1 antibodies or GPIb antibodies prior to UVB-hPLT injections, and protein concentration was determined. The protein concentration in BALF was reduced by 23% on average when mouse neutrophils were depleted (P<0.05, Figure 8), and reduced by 66% on average when mPLTs were depleted (P<0.01, Figure 8).

BALF protein content in the aspirin treated group was also significantly reduced, by 54% on average, when compared with DMSO treated control mice (Figure 8).

The above data indicate that depletion of mouse neutrophils or platelets, or aspirin treatment of hPLTs *in vitro* all protected mice from pulmonary vascular permeability change, a hallmark of ALI

## Discussion

Platelets have been shown to accumulate in the lungs of patients with ALI [21], and experimental animal models have further delineated the participation of platelets in ALI [3], [22]. Using our previously decribed SCID mouse model of ALI, we further dissected the role of the host mPLTs and infused hPLTs, and provided the first evidence for a contribution of host platelets in the development of ALI through recruitment of infused hPLTs. Our data show that retention of hPLTs in the lung of SCID mice pre-treated with LPS is largely depended on the initial lung sequestration of host mPLTs. Depletion of mPLTs eliminated the accumulation of UVB-treated hPLTs in the lung by 96%, and protected the mouse against UVB-hPLTs mediated ALI as evidenced by a 66% reduction of protein in the BALF. However mPLT depletion did not alter the neutrophil count in the lung. Conversely, neutrophil depletion resulted in a modest reduction (−20%) of UVB-hPLT retention in the lung and reduced the protein content in BALF by 23%, in proportion to the reduced PLT retention. These observations indicate that both host mPLTs and neutrophils participated in the development of ALI, although each to a different extent. We also show that aspirin treatment *in vitro*, which inhibited hPLTs aggregation, effectively abolished UVB-hPLTs accumulation in the lung and prevented ALI.

Adhesion between neutrophils and endothelial cells is thought to play a key role in the progression of ALI [23]. Changes in the lung become evident 2–4 hours after LPS administration and include neutrophil deformation and entrapment of neutrophils in the pulmonary circulation [24], [25]. Using the SCID/LYS-eGFP mice, we observed significant recruitment of neutrophils to the lungs 2 hours after intravenous injection of LPS. The recruited neutrophils exhibited morphological changes, extending lamellipodia or filopodia (Figure 2A-d to f). These neutrophils do recruit a small percentage of UVB-hPLTs to the lung although the majority of hPLTs sequestered in the lung were not bound to neutrophils (Figure 6B).

One interesting difference between our model and a number of recently published experimental mouse models of ALI [3], [7], [22] is that we did not observe significant increase in neutrophil accumulation in the lungs of the LPS+UVB PLT group as compared to the LPS+Ctrl PLT group even though only the LPS+UVB PLTs were associated with ALI. Another interesting finding is that neutrophil retention in the lung is independent of host platelets and persists when platelets were depleted. This indicates that neutrophils are not recruited by the platelets accumulating in the lung.

Overall our data suggest several possibilities for a mechanism of ALI associated with UVB-platelets: a) the UVB-hPLT caused ALI is less neutrophil dependent than that caused by allo-antibodies [3] or acid aspiration [22], both of which required an increase in neutrophils in the lungs for ALI to develop, b) neutrophils in our model participated in the development of ALI by increasing their proteolytic activities or by redistribution in the lungs without additional recruitment and 3) other cell types, such as the macrophages, are involved in mediating the damage to endothelial cells [26]. Macrophages were shown to produce proinflammatory cytokines and matrix metalloproteinases (MMPs), both of which can play an important role in the development of ALI [27], [28]. An alternative mechanism for ALI in our model is that partially activated UVB-hPLTs bind to the localized host platelets and build onto established platelet aggregates to form hemostatic plugs that occlude the microvessels in the lung. These occlusions can cause direct damage to the endothelial cells, as well as indirect damage, such as increased hydrostatic pressure proximal to the occlusion and hypoxic changes distal to the occlusion. Collectively these events could cause increased vascular permeability and the formation of a combination of transudate and exudate in the aveoli.

P-selectin expression on PLTs was shown to be elevated after UVB treatment [14]. We tested whether P-selectin mediated retention of hPLTs in the lung by injecting TRAP-activated hPLTs into SCID mice. P-selectin expression on TRAP (4 uM)-activated platelets was around 50%, similar to UVB treated platelets. We found that compared to the UVB treated hPLTs, 77% less hPLTs accumulated in the lung in TRAP-activated PLT group, a value comparable to Ctrl PLT group. This indicates that P-selectin was not the molecule that mediated UVB-hPLTs accumulation in the lung.

Our model has the advantage of being able to separate the contributions of host platelets and transfused platelets based on the species difference. hPLTs can be visualized by the human-specific anti-CD41 antibody, which does not react with mPLTs. Meanwhile, mPLTs can be visualized separately by endogenous, platelet-specific YFP expression from CD41-YFP mice. In addition, mPLTs can be depleted by GP1b antibodies, while infused hPLTs remain in circulation since mouse GP1b antibodies we used do not react with hPLTs.

We are aware of the limitation of our model system which includes a significant interspecies difference in structure and/or isoforms of proteins expressed on mPLTs versus hPLTs that could affect hPLT adhesion and aggregation [29] in a mouse model. Ultrastructurally, the similarities are much greater than the differences [30]. Most hPLT agonists, such as arachidonate, thrombin, collagen and ADP, can be used to activate mPLTs [31], [32]. The glycoprotein complexes, the platelet receptors in adhesion and aggregation that lead to a platelet plug, are conserved between mouse and human. Functionally, hPLTs were found to be active in mouse circulation. Human PLTs, produced upon transplantation of human cord blood progenitor cells [33] or human stem cells [34] can incorporate into mouse thrombi formed *ex vivo* or *in vivo*. This mouse-human platelets interaction was specific and, like in human platelet-to-platelet interaction, was mediated by glycoprotein GPIIb/IIIa [33]. The SCID mouse model has been used previously to evaluate hPLTs products [14], [20] and in xenotransplatation studies of human tissues [35], [36]. Therefore the use of this SCID mouse model allows a close recapitulation of the cellular events in human transfusion patients, before a more ideal, “humanized” mouse model is available.

These findings from our model of ALI will help define the potential clinical consequences of infusing platelets activated and/or damaged by UVB light. Certain medical conditions, such as sepsis modeled by the LPS injection into mice, may mediate redistribution of endogenous platelets and neutrophils in patients. Infusion of UVB treated or similarly damaged platelets could result in an interaction between the endogenous platelets and transfused platelets leading to accumulation of platelets in the lung. The physiologic response to this accumulation could lead to localized tissue damage and lung injury. Because the response of the organism is likely to be complex involving various cell types, cytokines and proteases, it remains necessary to study the whole organism and to use animal models to investigate different mechanisms and therapeutic strategies for UVB platelets induced ALI.

## Supporting Information

Figure S1 Analysis of anti-human CD41 antibody cross species reactivity by immunofluorescence. **A:** hPLT smear using PRP stained with anti-hCD41 antibodies (HIP8 clone). **B and C:** Blood smears from CD41-YFP mice, stained with either anti-GFP antibodies (B) or with a mixture of anti-hCD41 antibodies and anti-GFP antibodies (C). Anti-hCD41 antibodies show specific reactivity against hPLTs (A), but not against mPLTs (C). Anti-hCD41 antibodies were stained in red and anti-GFP antibodies were stained in green. The composite images are representatives of 3 independent experiments that showed the same result. All images were taken using a Zeiss 710 laser scanning confocal microscope, with a Plan-Apochromat 63×/NA1.4 oil objective. n = 3.
(TIF)

Figure S2 GPIb antibodies show specificity against mouse platelets. **A**: Flow cytometic analysis of mouse whole blood (WB, A-a, a′) and human apheresis platelets (A-b, b′) using GPIb antibodies and CD41 antibodies. mPLT and hPLT was gated on FSC versus SSC dot blot (A-a, b) and gated cells were analyzed for GPIb-FITC expression (A-a′, b′). mCD41-PE or hCD41-PE was used to label all mPLTs or hPLTs. **B**: Immunofluorescence staining with GPIb antibodies on mouse whole blood smear (B-a, a′) and human apheresis platelet smear (B-b, b′). Anti-GPIb antibodies (in red) specifically label mouse platelets (B-a), which also stain positive for mCD41 antibody (in green, B-a′). No human platelets stain positive for GPIb antibodies (B-b) and these human platelets stain positive for hCD41 antibody (in green, B-b′). The plots and the composite image are representatives of 2 independent experiments that showed the same result. All confocal images were taken using a Zeiss 710 laser scanning confocal microscope, with a Plan-Apochromat 63×/NA1.4 oil objective.
(TIF)

Figure S3 GPIb antibodies efficiently depleted mouse platelets. SCID/CD41-eYFP mice were pretreated with GPIb antibodies (2 mg/kg, i.v.) 4 hours before LPS administration (3 mg/kg, i.v.). Peripheral blood platelets were depleted 4 hours after receiving GPIb antibodies (c, d vs a, b), visualized by endogenous e-YFP fluorescence on blood smears from SCID/CD41-eYFP mice. Platelets in the lungs were also depleted by GPIb antibodies (g, h vs e, f), visualized by endogenous e-YFP fluorescence on frozen lung sections from SCID/CD41-eYFP mice. n = 5.
(TIF)

Figure S4 CMTMR labeling doesn't activate hPLTs. **A–C**: Flow cytometric analysis of Ctrl hPLT or UVB-treated hPLT, before (C) or after (B) CMTMR labeling. hPLTs were gated on SSC vs. FSC dot blot (A). The gated hPLTs were analyzed for % CD62P (P-selectin) positive cells. B-a,b: dot plots of unstained samples before CMTMR labeling. B-c,d: dot plots of stained samples (with hCD41-FITC and hCD62P-PE antibodies) before CMTMR labeling. C-a′,b′: dot plots of unstained samples after CMTMR labeling. C-c′,d′: dot plots of stained samples (with hCD41-FITC and hCD62P-PE antibodies) after CMTMR labeling. % CD62P (P-selectin) positive cells were also analyzed using CD62P-FITC antibody (plots not shown). The plots are representatives of 3 independent experiments and the data was summarized in D. ** p<0.01.
(TIF)
