# Supplementary figures and images for: Host Platelets and, in Part, Neutrophils Mediate Lung Accumulation of Transfused UVB-Irradiated Human Platelets in a Mouse Model of Acute Lung Injury
Source: PLoS One. 2012 Sep 19;7(9):e44829. doi: 10.1371/journal.pone.0044829 (PMC3446987; doi:10.1371/journal.pone.0044829)

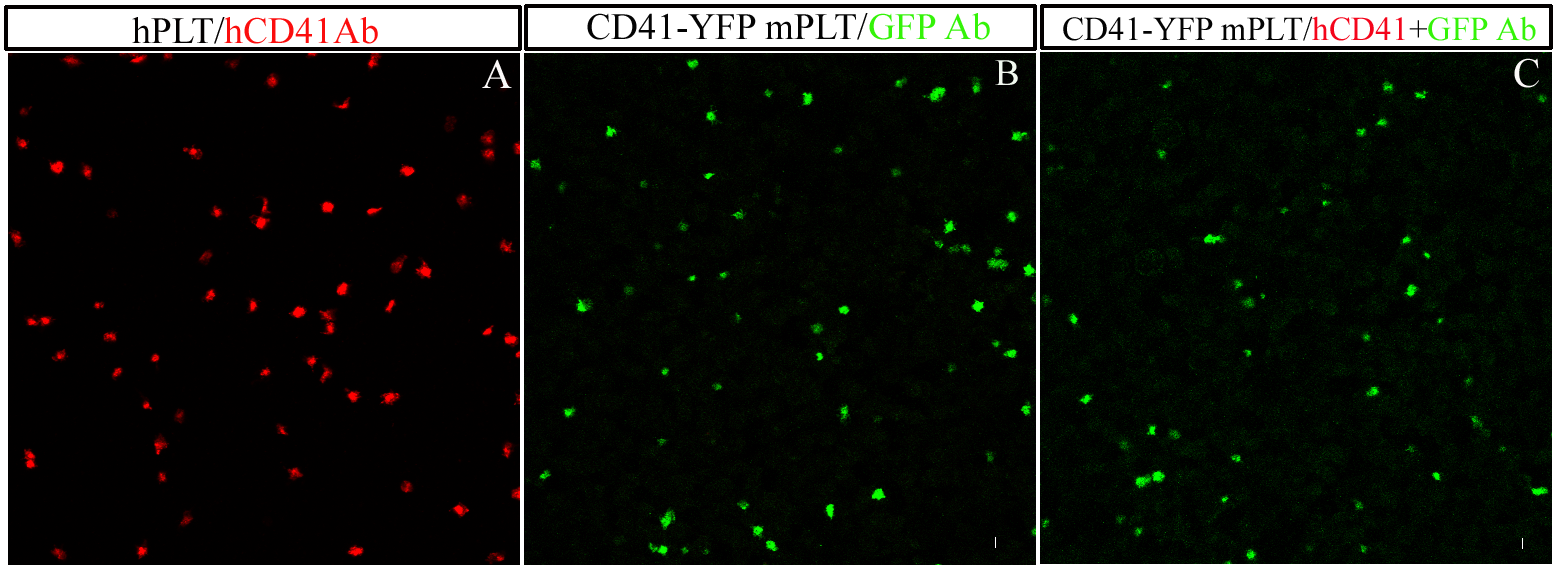

Supplement: Figure S1 — Analysis of anti-human CD41 antibody cross species reactivity by immunofluorescence. A: hPLT smear using PRP stained with anti-hCD41 antibodies (HIP8 clone). B and C: Blood smears from CD41-YFP mice, stained with either anti-GFP antibodies (B) or with a mixture of anti-hCD41 antibodies and anti-GFP antibodies (C). Anti-hCD41 antibodies show specific reactivity against hPLTs (A), but not against mPLTs (C). Anti-hCD41 antibodies were stained in red and anti-GFP antibodies were stained in green. The composite images are representatives of 3 independent experiments that showed the same result. All images were taken using a Zeiss 710 laser scanning confocal microscope, with a Plan-Apochromat 63×/NA1.4 oil objective. n = 3. (TIF) [file pone.0044829.s001.tif]

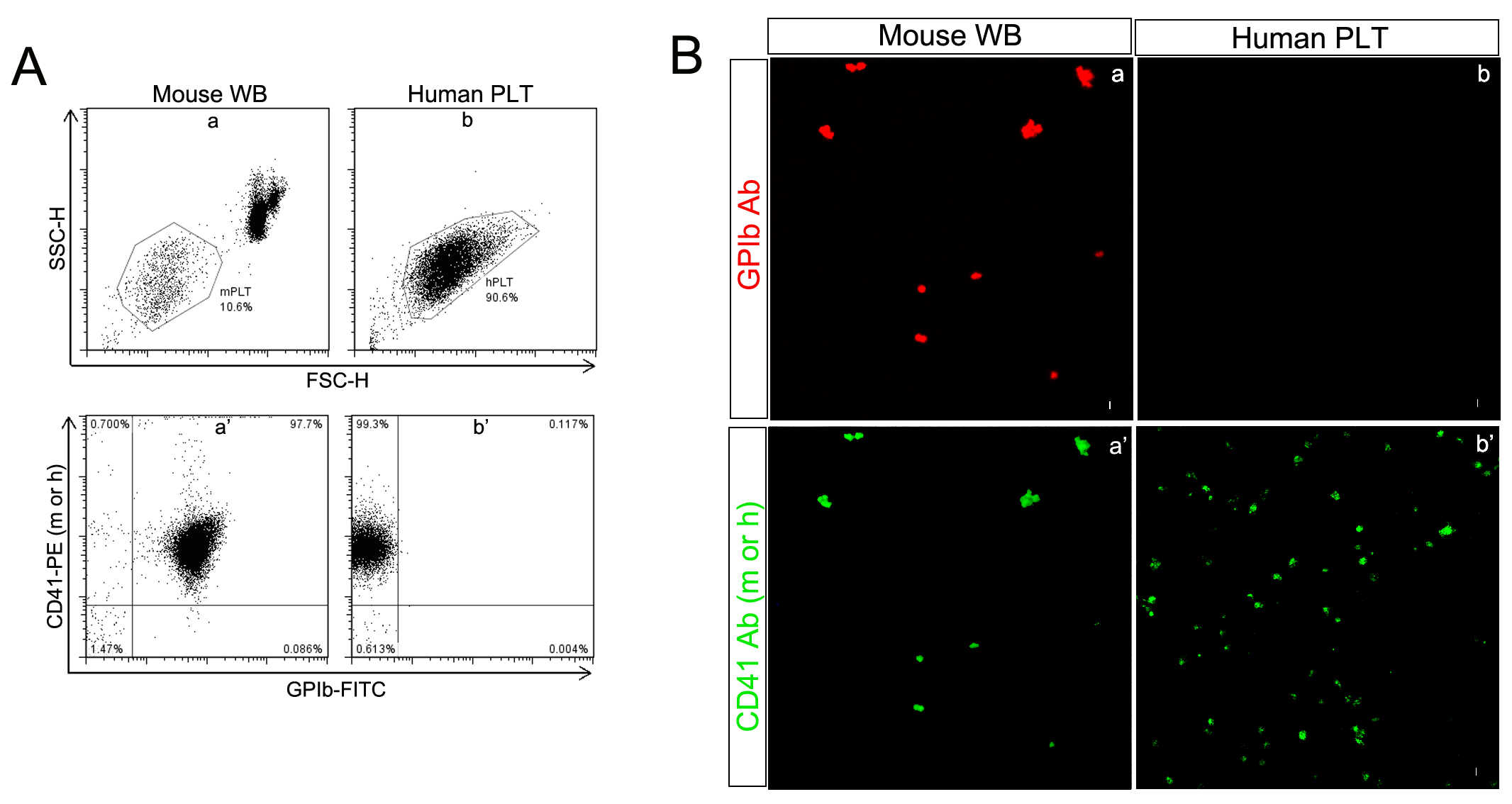

Supplement: Figure S2 — GPIb antibodies show specificity against mouse platelets. A: Flow cytometic analysis of mouse whole blood (WB, A-a, a′) and human apheresis platelets (A-b, b′) using GPIb antibodies and CD41 antibodies. mPLT and hPLT was gated on FSC versus SSC dot blot (A-a, b) and gated cells were analyzed for GPIb-FITC expression (A-a′, b′). mCD41-PE or hCD41-PE was used to label all mPLTs or hPLTs. B: Immunofluorescence staining with GPIb antibodies on mouse whole blood smear (B-a, a′) and human apheresis platelet smear (B-b, b′). Anti-GPIb antibodies (in red) specifically label mouse platelets (B-a), which also stain positive for mCD41 antibody (in green, B-a′). No human platelets stain positive for GPIb antibodies (B-b) and these human platelets stain positive for hCD41 antibody (in green, B-b′). The plots and the composite image are representatives of 2 independent experiments that showed the same result. All confocal images were taken using a Zeiss 710 laser scanning confocal microscope, with a Plan-Apochromat 63×/NA1.4 oil objective. (TIF) [file pone.0044829.s002.tif]

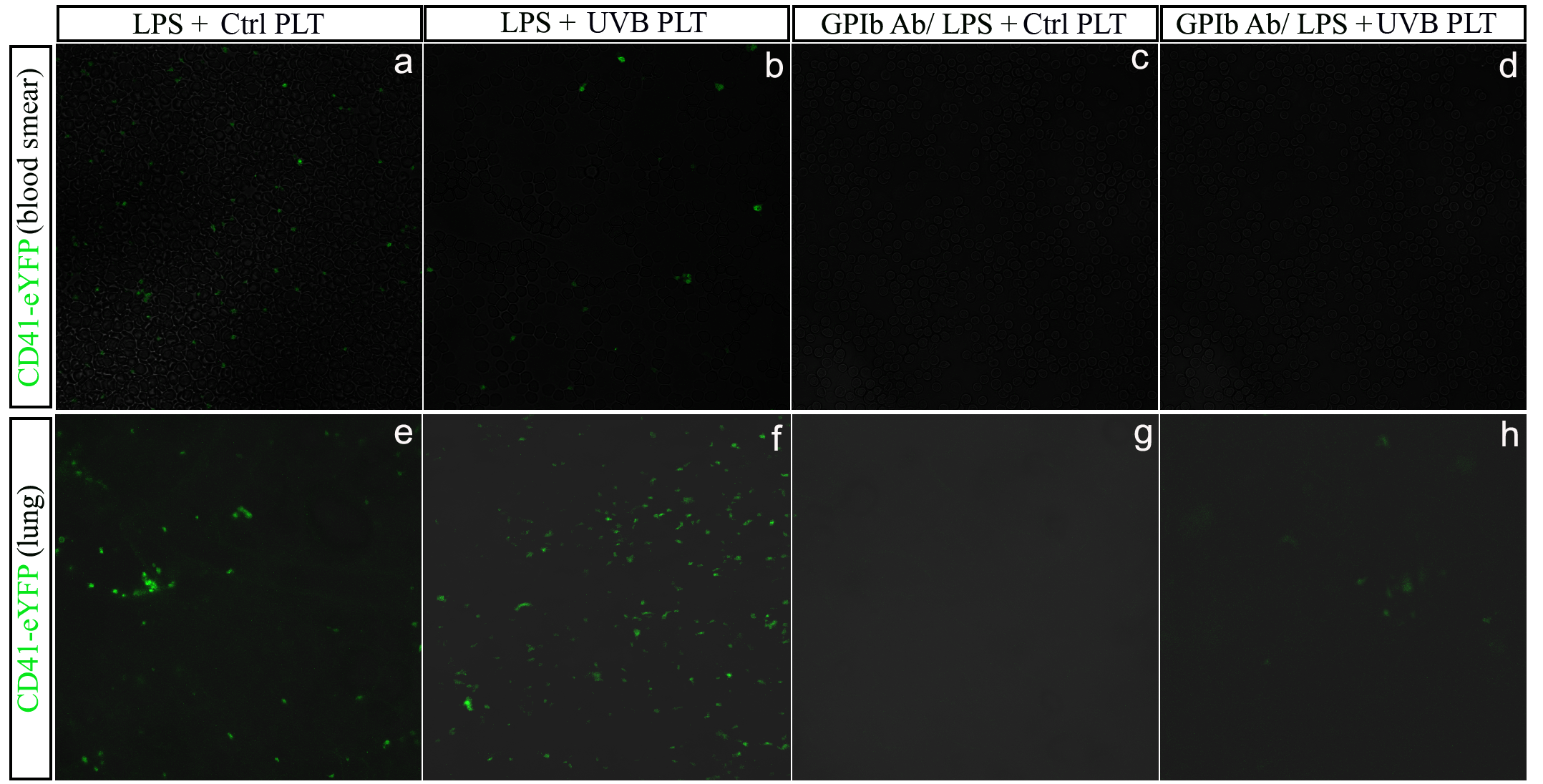

Supplement: Figure S3 — GPIb antibodies efficiently depleted mouse platelets. SCID/CD41-eYFP mice were pretreated with GPIb antibodies (2 mg/kg, i.v.) 4 hours before LPS administration (3 mg/kg, i.v.). Peripheral blood platelets were depleted 4 hours after receiving GPIb antibodies (c, d vs a, b), visualized by endogenous e-YFP fluorescence on blood smears from SCID/CD41-eYFP mice. Platelets in the lungs were also depleted by GPIb antibodies (g, h vs e, f), visualized by endogenous e-YFP fluorescence on frozen lung sections from SCID/CD41-eYFP mice. n = 5. (TIF) [file pone.0044829.s003.tif]

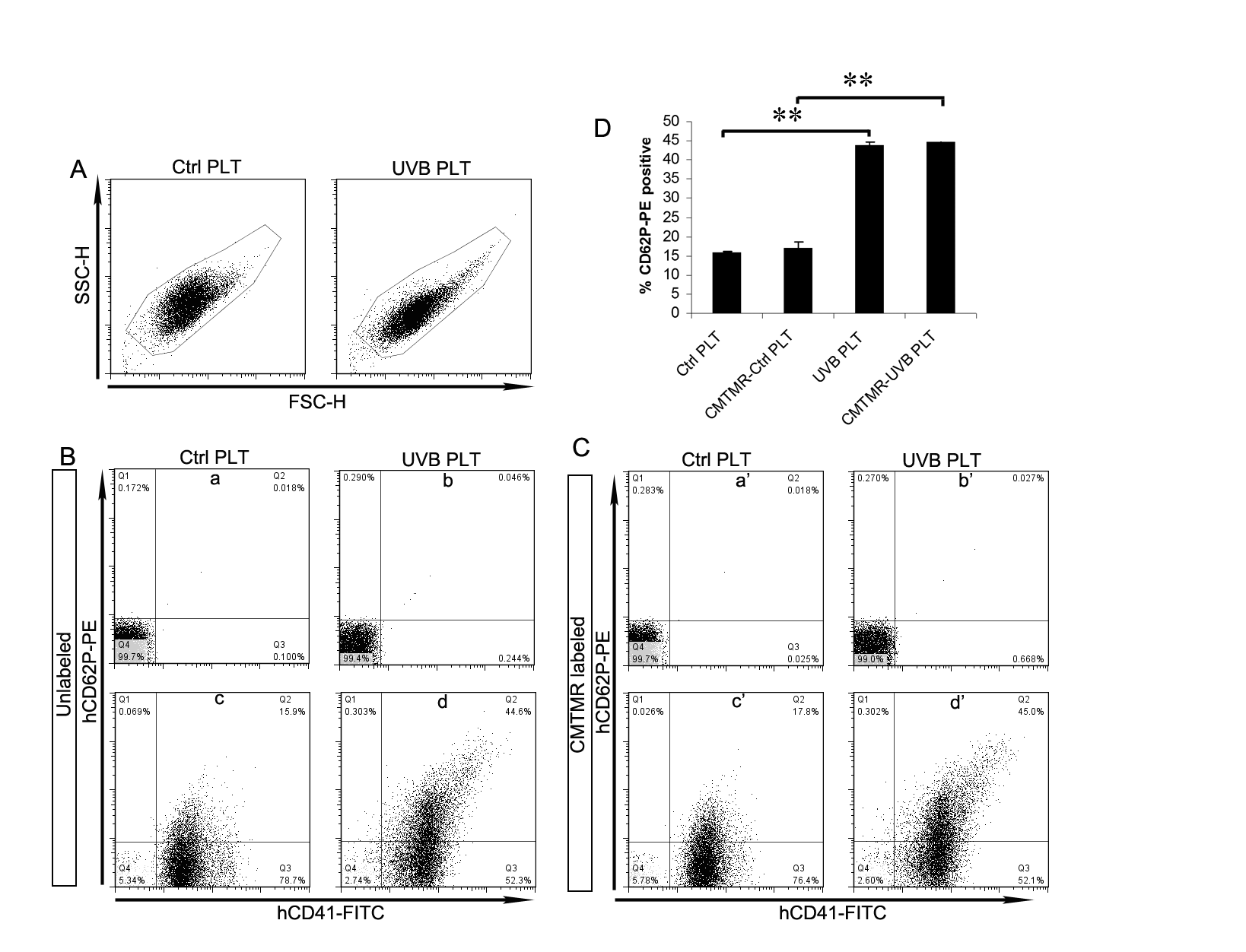

Supplement: Figure S4 — CMTMR labeling doesn't activate hPLTs. A–C: Flow cytometric analysis of Ctrl hPLT or UVB-treated hPLT, before (C) or after (B) CMTMR labeling. hPLTs were gated on SSC vs. FSC dot blot (A). The gated hPLTs were analyzed for % CD62P (P-selectin) positive cells. B-a,b: dot plots of unstained samples before CMTMR labeling. B-c,d: dot plots of stained samples (with hCD41-FITC and hCD62P-PE antibodies) before CMTMR labeling. C-a′,b′: dot plots of unstained samples after CMTMR labeling. C-c′,d′: dot plots of stained samples (with hCD41-FITC and hCD62P-PE antibodies) after CMTMR labeling. % CD62P (P-selectin) positive cells were also analyzed using CD62P-FITC antibody (plots not shown). The plots are representatives of 3 independent experiments and the data was summarized in D. ** p<0.01. (TIF) [file pone.0044829.s004.tif]
